# Glioblastoma Mimicking an Arteriovenous Malformation

**DOI:** 10.3389/fneur.2013.00144

**Published:** 2013-09-30

**Authors:** Arjun Khanna, Andrew S. Venteicher, Brian P. Walcott, Kristopher T. Kahle, Daniel A. Mordes, Christopher M. William, Zoher Ghogawala, Christopher S. Ogilvy

**Affiliations:** ^1^Harvard Medical School, Boston, MA, USA; ^2^Department of Neurosurgery, Massachusetts General Hospital, Boston, MA, USA; ^3^Department of Pathology, Massachusetts General Hospital, Boston, MA, USA; ^4^Department of Neurosurgery, Lahey Hospital and Medical Center, Tufts University School of Medicine, Burlington, MA, USA

**Keywords:** glioblastoma, astrocytoma, subarachnoid hemorrhage, intraparenchymal hemorrhage, angiography, arteriovenous malformation

## Abstract

Abnormal cerebral vasculature can be a manifestation of a vascular malformation or a neoplastic process. We report the case of a patient with angiography-negative subarachnoid hemorrhage (SAH) who re-presented 3 years later with a large intraparenchymal hemorrhage. Although imaging following the intraparenchymal hemorrhage was suggestive of arteriovenous malformation, the patient was ultimately found to have an extensive glioblastoma associated with abnormal tumor vasculature. The case emphasizes the need for magnetic resonance imaging to investigate angiography-negative SAH in suspicious cases to rule out occult etiologies, such as neoplasm. We also discuss diagnostic pitfalls when brain tumors are associated with hemorrhage and abnormal vasculature.

## Introduction

Subarachnoid hemorrhage (SAH) results from both traumatic and non-traumatic etiologies. While vascular malformations such as arteriovenous malformations (AVM) and aneurysms account for the majority of non-traumatic SAH, no specific cause can be found in many cases. Even with catheter-based diagnostic cerebral angiography, no causative etiology is seen in 15–30% of cases (1, 2). Among non-traumatic, non-aneurysmal SAH, neoplasms represent a minority (1–3%) of all cases (1). Herein, we report the case of a patient with angiography-negative SAH who re-presented 3 years later with rebleeding from what appeared to be *de novo* AVM. The patient was ultimately found to have a glioblastoma associated with tumor-related vasculature mimicking an AVM. We discuss challenges in the diagnosis of glioblastoma in the setting of intracranial hemorrhage (ICH) and tumor-associated vascular abnormalities that resemble AVM. We also argue for the use of contrast-enhanced magnetic resonance imaging to investigate the etiology of angiography-negative SAH when warranted based on clinical suspicion.

## Case Report

**Figure 1:**
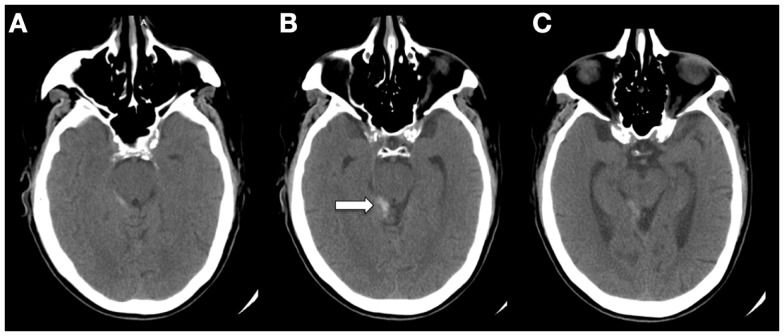
**CT (A–C) demonstrating SAH in the quadrigeminal and supracerebellar cisterns (arrow)**. The image was taken 48–64 h after minor trauma to the head while the patient was anticoagulated with warfarin. Although this is not a classic traumatic location for SAH, redistribution may have occurred between the time of trauma and when the image was taken.

A 53-year-old male anticoagulated with warfarin for stroke prevention (atrial fibrillation) presented with blurry vision and headache that had progressively worsened over the course of 3 days following minor trauma to the head. Computed tomography (CT) demonstrated SAH in the right quadrigeminal and supracerebellar cisterns (Figure 1). Catheter-based angiography showed no evidence of intracranial aneurysm, AVM, arteriovenous (A-V) fistula, or other vascular malformation. The angiography-negative SAH was initially attributed to traumatic etiology. The patient developed renal insufficiency, required dialysis, and was discharged to home.

**Figure 2:**
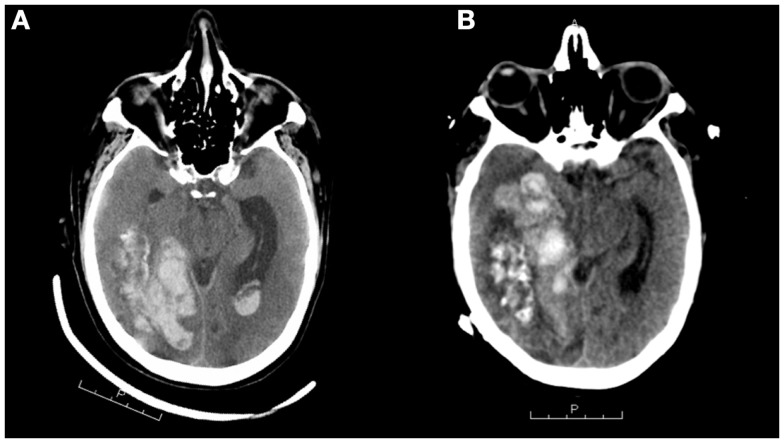
**CT scans (A,B) demonstrating intracranial hemorrhage 3 years after initial SAH**. The left scan shows a large right temporal hematoma that was emergently evacuated via craniotomy. The right scan, taken 1 month later, shows extensive intraparenchymal hemorrhage centered in the right temporal lobe extending into the right parietal and occipital lobes measuring up to 7.8 cm × 3.7 cm. There is bleeding into the ventricular system. Note the mass effect, causing midline shift toward the left of 7 mm and uncal herniation.

**Figure 3:**
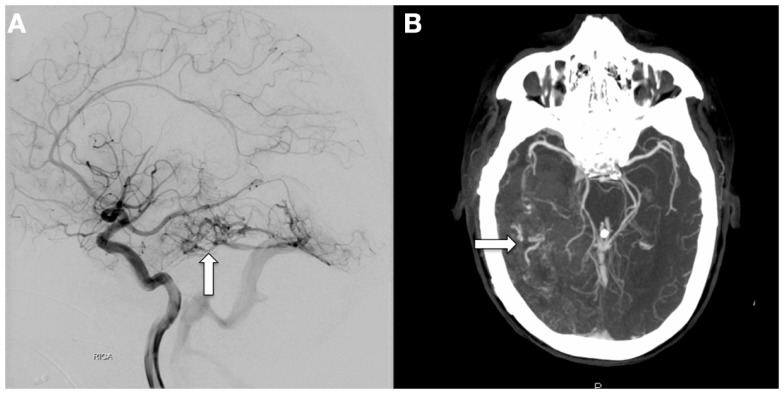
**Catheter-based, diagnostic cerebral angiogram (A) and CT-angiogram (B) taken following the CT scans on the left and right of Figure 2, respectively, showing a large tangle of abnormal vessels centered in the right temporal lobe covering an area of at least 7.8 cm in the AP dimension (arrows)**. The right P2 segment appears to feed a portion of this vascular malformation, and an enlarged vein appears to drain into the basal vein of Rosenthal. These findings are suggestive of AVM.

Three years later, following an episode of hemodialysis, the patient developed acute onset of unresponsiveness, lack of right pupillary response to light, and left-sided weakness. CT imaging demonstrated a large right temporal hematoma (Figure 2) that was emergently evacuated via craniotomy. CT and catheter angiography demonstrated a large region of abnormal vasculature in the right temporal, parietal, and occipital areas with a slow A-V shunt, suggestive of an AVM (Figure 3). The lesion was treated with Onyx-18 liquid embolic agent (eV3 Neurovascular Inc., Irvine, CA, USA) with partial obliteration of the A-V shunt. He was discharged to an inpatient rehabilitation hospital and prescribed anticoagulation for lower extremity deep vein thrombosis.

Three weeks later, he again became acutely unresponsive with left-sided weakness. A head CT demonstrated new and extensive intraparenchymal hemorrhage centered in the right temporal lobe, extending into the right parietal, and occipital lobes measuring up to 7.8 cm × 3.7 cm (Figure 2). There was hemorrhage in the ventricular system, along with SAH in the area of the right parietal lobe. There was an extensive mass effect from the intraparenchymal component of the hemorrhage, causing midline shift of 7 mm, and right uncal herniation.

His anticoagulation was reversed emergently and he was stabilized in the intensive care unit. A CT-angiogram demonstrated a large region of abnormal vasculature centered in the right temporal lobe (Figure 3). The right posterior cerebral artery appeared to feed a portion of the lesion. Interpretation of the vascular imaging was complicated by the mass effect associated with the intraparenchymal hemorrhage, which distorted normal anatomy. While in the intensive care unit, the patient began experiencing seizures, prompting repeated imaging. A CT scan demonstrated the development of a new intraparenchymal hemorrhage within the region of the right basal ganglia, as well as an SAH in the right frontal lobe with significant associated edema and resultant 2 cm midline shift with uncal herniation. Given the significant risks of further neurosurgical intervention, poor neurological state, and low likelihood of recovery, the family elected to transition his goals of care to comfort measures only. The patient expired 5 days later.

**Figure 4:**
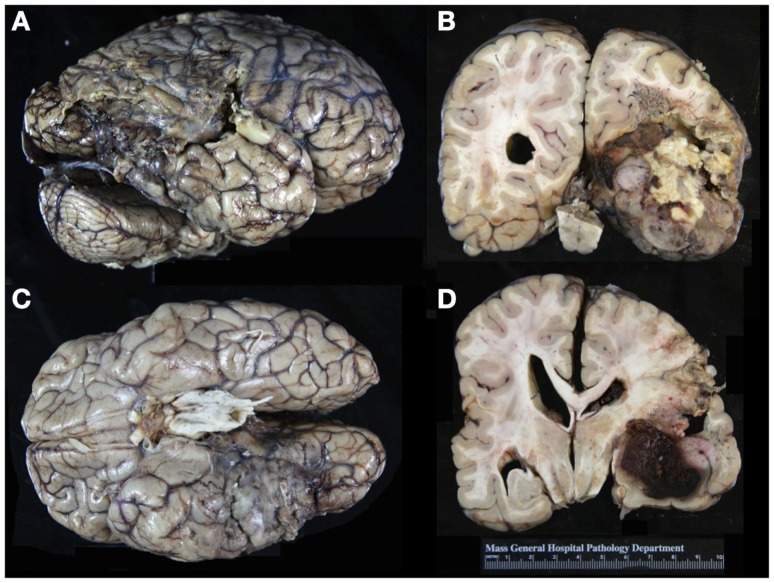
**Gross appearance of the patient’s brain on autopsy**. Note the prominent vasculature over the right superior cerebellum and inferior surface of the right temporal lobe **(A,C)**. There was softening of the cortex over a 12 cm × 6 cm area spanning the right posterior temporal, right inferior parietal, and right anterior occipital regions. In the coronal sections, note the large area of yellow-white necrosis adjacent to the hemorrhage. There is tan to gray-white discoloration of the cortex in the right temporal and parietal lobes, reflecting extensive involvement by tumor**(B,D)**.

Autopsy revealed no evidence for a vascular malformation; rather, an extensive glioblastoma involving the right parietal, occipital, and temporal lobes with significant associated hemorrhage and necrosis was found (Figure 4). The tumor contained many medium-caliber sclerotic vessels and large areas of necrosis. Grossly, there was increased prominence of veins over the right superior cerebellum and the inferior surface of the right temporal lobe. There were contusions in the right orbitofrontal region and bilateral temporal tips, and increased vascular markings over the surface of the right inferior temporal gyrus. A softening of the cortex (12 cm × 6 cm) spanning the right posterior temporal, right inferior parietal, and right anterior occipital regions was noted.

**Figure 5:**
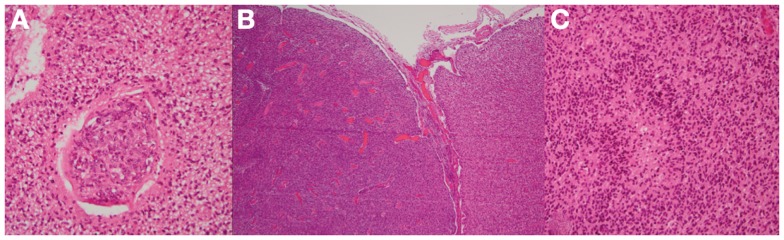
**Microscopic examination demonstrating glioblastoma with neovascularization (A,B)**. The glioblastoma extensively infiltrates and replaces the entire cortical band **(B)**. Tumor vasculature is structurally different from normal vessels and is generally more prone to hemorrhage. There was a classic pseudopalisading pattern of necrosis that is characteristic of glioblastoma**(C)**.

Histologically, this was a WHO grade IV glioblastoma with extensive infiltration of the white matter centered in the right temporal lobe and involving the parietal and occipital lobes. Classic pseudopalisading necrosis, along with frequent mitotic figures, microvascular proliferation, and reactive gliosis involving the cortex and white matter were noted (Figure 5). There were large areas of geographic necrosis, and scattered medium to large-sized vessels with intraluminal thrombi.

## Discussion

This patient first presented with an angiography-negative SAH in the quadrigeminal and supracerebellar cisterns with a history of mild trauma to the head. Although non-aneurysmal SAH has a generally better prognosis than aneurysmal SAH, non-aneurysmal SAH encompasses multiple disease processes, the prognosis for which can vary (2). Among these is the perimesencephalic SAH that has elusive etiology but excellent prognosis, with minimal risk of rebleeding (3, 4). Longitudinal studies of outcomes following perimesencephalic SAH find no increased risk of future SAH, no residual functional deficits after 3 months, and no change in overall life expectancy in these cases (5–7). Angiography-negative hemorrhage in the quadrigeminal cistern has been proposed as a variant of perimesencephalic SAH with a similarly benign prognosis, although this categorization has been debated (8). Other non-aneurysmal causes of SAH include coagulopathy, hypertension, infection, venous occlusive disease, occult trauma, drug abuse, and cerebral neoplasia (9). Tumors are usually apparent on CT; however, at least one other case of occult glioma presenting solely as angiogram-negative SAH has been reported (10).

As there was no evidence of neoplasm on CT and the presented patient had a history of mild trauma while on warfarin, it was suspected that the original presentation of SAH 3 years prior had a traumatic etiology exacerbated by coagulopathy. The SAH was not in a classic traumatic location, although a redistribution phenomenon is known to occur when significant time (48–64 h) elapses between trauma and imaging. It is uncertain whether the patient harbored a hemorrhagic tumor 3 years prior to his most recent presentation, although it remains a possibility. There has been one other report of glioblastoma presenting as a suspected traumatic ICH, in which the authors emphasize the importance of workup for possible neoplastic etiology when hemorrhage occurs in an abnormal site (11). However, glioma-related hemorrhage is almost exclusively intraparenchymal, and only rarely enters the subarachnoid space (12, 13).

Three years later, the patient re-presented with an extensive intraparenchymal hemorrhage. Brain tumors represent 0.9–11% of spontaneous ICH, and between 3 and 5% of intracranial neoplasms cause large hemorrhage; the incidence approaches 15% if subclinical and microscopic hemorrhage is considered (14). The incidence of ICH is greater in faster-growing tumors with irregular vasculature (15). Glioblastoma is the most common primary intracranial neoplasm, accounting for 15–20% of all cases, and is the most frequent cause of hemorrhage among the primary brain tumors (16).

Glioblastoma-associated hemorrhages are usually located deep within the cerebral hemispheres, corpus callosum, or basal ganglia. Several hypotheses have been proposed to explain the pathological basis of glioma-associated hemorrhage. Hemorrhage within the tumor may be caused by disruption of vessels traversing the necrotic core. Tumor erosion of cerebral vessels at proliferating margins may cause thinning, rupture, distortion, compression, or aneurysm of normal brain vasculature. Tumor capillaries may be structurally deficient and susceptible to bleed (15, 17). Some have suggested that abnormal gliovascular interactions within tumor vessels make them more susceptible to rupture (11). Peritumoral vasculature may become similarly abnormal (12). Finally, intratumoral suppression of the tissue-factor dependent coagulation cascade may predispose tumor vasculature to hemorrhage (18).

Glioblastoma or other intracranial malignancy presenting as ICH poses a diagnostic dilemma, because recognizing the neoplastic lesion is not always possible with CT or CT-angiography. Indeed, in a retrospective study of 50 cases of spontaneous ICH caused by cerebral neoplasms, there was no preoperative radiological suspicion of neoplasm in half of cases (19). Nevertheless, there are some distinctive features of tumor-related ICH on CT that may help guide workup. Irregular shape and atypical location of an ICH may indicate neoplastic etiology (19), including cases of ICH precipitated by trauma (11). Heterogeneous appearance and multiple hemorrhage locations may also suggest a tumor (20). Enhanced peritumoral vascularization at tumor margins and vascular erosion of tissue around the tumor likely contribute to ring-like enhancement around the lesion that is particularly apparent on contrast-enhanced CT (21). Perilesional edema may be an important finding, because it is rare in acute ICH but common in expanding, space-occupying lesions (22, 23).

In the case presented, angiography to investigate the etiology of ICH demonstrated a large region of irregular vasculature (ultimately proving to be spatially coincident with glioblastoma) that demonstrated slow A-V shunting. The combination of ICH and abnormal vasculature with A-V shunting on angiography was highly suggestive of AVM in this patient. The normal angiogram obtained 3 years prior to his admission was not inconsistent with a diagnosis of AVM, as *de novo* AVMs are known to occur (24). In addition to vascular malformations, there can be significant A-V shunting in many brain tumors that can be visualized on angiography (25, 26). The relationship between development of glioblastoma and AVM-like angiopathy is unclear, but pro-angiogenic factors secreted by growing malignancies are suspected to play an important role in the development of vascular abnormalities, including A-V shunting, in glioblastoma and other cerebral neoplasms (27).

The radiographic features that would enable differentiation between AVM and glioblastoma are frequently obfuscated in the setting of acute hemorrhage. For example, a ruptured AVM with hematoma formation can exhibit a mass effect, making it difficult to distinguish from a tumor on CT. In one study, 33 of 60 cases of ruptured AVMs demonstrated significant mass effect (28).

Although rare, there have been a few reports of glioblastoma mimicking or associated with a vascular lesion in which initial imaging does not clearly distinguish between the two. In some cases, initial imaging indicates hemorrhage of uncertain etiology (11, 29–31); in others, CT or CT-angiogram is specifically suggestive of AVM (32, 33), as in the presented case. Some reports describe coincidence of GBM and AVM in the same patients, even occasionally without spatial overlap between the lesions (34, 35). In all of these cases, histopathological diagnosis ultimately confirmed GBM. One report highlights the importance of including perilesional tissue in biopsy whenever possible, describing a case in which diagnosis of GBM was initially missed because only a small minority of sections from a vascular-appearing lesion showed evidence of neoplasm (36). Taken together, these cases and ours highlight the importance of considering neoplasm in the differential diagnosis of ICH with uncertain etiology, and the need for careful monitoring when initial workup in inconclusive.

The anatomical coincidence of the first SAH and the large glioblastoma discovered 3 years later suggests that the etiology of the SAH may have been neoplastic. We suspect that a follow-up contrast-enhanced MRI to further investigate the cause of the patient’s SAH may have uncovered a malignant lesion, or at least would have been useful in narrowing our differential for the etiology. Indeed, in one study, MRI following angiogram-negative ICH provided new, valuable diagnostic information in 36% of cases (37).

Additionally, better differentiation between AVM and glioblastoma would have been possible on MRI when the patient re-presented 3 years later. AVMs have characteristic low signal intensity on T1-weighted images and high signal intensity with signal voids on T2-weighted images, and can be enhanced by contrast. Glioblastoma is characterized by poorly circumscribed infiltration associated with significant perilesional edema. In contrast-enhanced MRI, glioblastoma usually shows heterogeneous enhancement. However, even with MRI, early findings of glioblastoma may resemble non-neoplastic lesions (31).

We propose that contrast-enhanced MRI may be useful for investigating the etiology of non-aneurysmal SAH, particularly for distinguishing benign causes like perimesencephalic SAH from more harmful ones, such as neoplasm. Given the known association between glioblastoma and AVM-like angiopathy, we also emphasize the importance of workup for possible neoplastic etiology in the setting of ICH and AVM-like findings on angiography, in which MRI may also play an important role. This case highlights the need for careful consideration of neoplasm in the differential diagnosis of non-aneurysmal SAH, and the potential pitfalls in the diagnosis of glioblastoma associated with abnormal vasculature.

## Conflict of Interest Statement

The authors declare that the research was conducted in the absence of any commercial or financial relationships that could be construed as a potential conflict of interest.
